# Combining Cognitive Training and Brain Stimulation: A Novel Approach to Address Disordered Eating and Antipsychotic‐Induced Weight Gain in People With Severe Mental Illness

**DOI:** 10.1111/eip.70275

**Published:** 2026-10-26

**Authors:** Mariana Lopes, Dina Monssen, Luiza Grycuk, Gemma Gordon, Fiona Gaughran, Iain C. Campbell, Ulrike Schmidt

**Affiliations:** ^1^ Centre for Research in Eating and Weight Disorders, Department of Psychological Medicine, Institute of Psychiatry, Psychology & Neuroscience King's College London London UK; ^2^ Department of Psychosis Studies, Institute of Psychiatry, Psychology & Neuroscience King's College London London UK; ^3^ South London and Maudsley NHS Foundation Trust Maudsley Hospital London UK

**Keywords:** approach bias modification training, binge eating, food craving, schizophrenia, transcranial direct current stimulation

## Abstract

**Background:**

Antipsychotic‐induced weight gain is a distressing side effect that most often occurs within the first 3–6 months of pharmacological initiation, significantly increasing cardiometabolic risk and mortality in individuals with severe mental illness.

**Aims:**

To assess the feasibility of reducing food craving and binge eating using Approach Bias Modification Training (ABMT) combined with real or sham Transcranial Direct Current Stimulation (tDCS).

**Method:**

In this double‐blind, parallel‐group, two‐arm, sham‐controlled trial, adults with schizophrenia/schizoaffective disorder on stable doses of antipsychotics were randomly allocated to a total of five ABMT sessions together with either real or sham tDCS administered over 3 weeks. Assessments were made at baseline (T0), post‐treatment (T1) and at a 2‐week follow‐up (T2). Feasibility outcomes included recruitment, attendance, retention, tDCS safety, acceptability, tolerability, and blinding. Secondary outcomes were changes in food craving, binge eating, approach bias to food, body mass index, and psychological symptoms.

**Results:**

Twenty‐eight participants were recruited (93% of target). Retention was high (T1 = 85.7% and T2 = 78.6%); blinding accuracy was 46.4%. Medium effect sizes favoured ‘ABMT + real tDCS’ for reducing binge eating (*d* = −0.6, 95% CI −1.5, 0.3), and ‘ABMT + sham tDCS’ for reducing food cravings (*d* = 0.5, 95% CI −0.4, 1.4). Within‐groups, only the ‘ABMT + real tDCS’ arm showed reduced approach bias to food and approach‐avoidance bias to food, and increased avoidance bias to food (all *p* < 0.05). This was followed by reduced binge eating episodes (*p* < 0.05). Both groups reported frequent binge‐eating episodes (‘ABMT + tDCS’ = 43%; ‘ABMT + sham tDCS’ = 28.6%).

**Conclusion:**

The initiation of antipsychotic therapy is a critical clinical window for early intervention to mitigate the long‐term metabolic and psychiatric consequences of antipsychotic‐induced weight gain. Combining ABMT + tDCS is acceptable and may help reduce binge eating in individuals on antipsychotics, with a potential synergistic effect between cognitive training and brain stimulation. A confirmatory trial is needed to assess its effectiveness and should include BED diagnosis as a stratification criterion.

**Trial Registration:** ISRCTN Identifier: 13280178

**Abbreviations**
ABMT: approach bias modification training
BIS‐11: Barratt Impulsiveness Scale
BRC: Biomedical Research Centre
CONSORT: consolidated Standards of Reporting Trials
DASS‐21: depression, anxiety and stress scale
dlPFC: dorsolateral prefrontal cortex
DSM‐5: diagnostic and statistical manual for mental disorders, Fifth edition
EDE‐Q: eating disorder examination questionnaire
F‐AAT: food approach/avoidance task
FCQ‐T‐r: food cravings questionnaire‐trait‐reduced
FCT: food challenge task
fMRI: functional magnetic resonance imaging
IoPPN: Institute of Psychiatry, Psychology and Neuroscience
ISRCTN: International Standard Randomised Controlled Trial Number
MoCA: Montreal cognitive assessment
NIHR: National Institute for Health Research
PANSS‐6: Positive and Negative Syndrome Scale
RCT: randomised controlled trial
REC: Research ethics committee
rTMS: repetitive transcranial magnetic stimulation
SLaM: South London and Maudsley NHS Foundation Trust
SNAPSI: Simplified Negative and Positive Symptoms Interview
SPIRIT: Standard Protocol Items: Recommendations for Interventional Trials
SRCT: stimulus response compatibility task
tDCS: transcranial direct current stimulation
VAS: visual analogue scale

## 1 Introduction

Antipsychotic‐induced weight gain (AIWG) is a distressing treatment side effect, associated with medication discontinuation, psychiatric relapse, cardiometabolic morbidity, and with premature mortality in individuals with severe mental illness (SMI) (Brown et al. 2000; Ballon et al. 2014; Manu et al. 2015; Speyer et al. 2021). Given that rapid weight gain usually occurs within the first 3–6 months of antipsychotic therapy, the initiation of treatment provides a critical clinical window for early intervention to mitigate long‐term metabolic and psychiatric consequences (Bushe et al. 2012; O'Mahony et al. 2026).

AIWG arises from alterations in central neurotransmitter systems, (including histaminergic, serotonergic, and dopaminergic pathways), as well as in peripheral hormonal signalling (Coccurello and Moles 2010; Kowalchuk et al. 2017). These result in increased appetite, reduced satiation and energy expenditure, and increased fat storage (Coccurello and Moles 2010; Kowalchuk et al. 2017), which can lead to food craving and related disordered eating behaviours, for example, binge eating, (Blouin et al. 2008) or to binge eating disorder (BED), overweight and obesity (Theisen et al. 2003; Carolan et al. 2024). Pharmacological treatments, including metformin and GLP‐1 receptor agonists, show moderate efficacy in preventing and managing AIWG (Carolan et al. 2024; Campforts et al. 2024; Fink‐Jensen and Correll 2025; Fitzgerald et al. 2022). However, they are associated with physical side effects (Carolan et al. 2024; Campforts et al. 2024; Fink‐Jensen and Correll 2025), safety concerns in the mental health population, high costs (Carolan et al. 2024) and weight regain after drug discontinuation (Wilding et al. 2022). People with SMI are reported to have higher calorie intake, particularly from energy‐dense foods, and lower energy expenditure (Manu et al. 2015), and adherence to dietary and lifestyle interventions is challenging and limited in this group (Manu et al. 2015; Fink‐Jensen and Correll 2025). Consequently, there is a need for strategies that target the underlying causes of AIWG, such as food cravings and binge eating, to complement lifestyle and pharmacological interventions (Alvarez‐Jiménez et al. 2010; Bonfioli et al. 2012).

Cognitive training and neuromodulation techniques are relatively accessible interventions that have shown the potential to modify food‐related behaviours in people with eating disorders (Brockmeyer et al. 2015a; Brockmeyer et al. 2019). Approach bias modification training (ABMT), a computer‐based form of cognitive training, aims to change implicit attention biases by training individuals to avoid a negative stimulus (e.g., high‐calorie food) and to direct their attention to a neutral or positive stimulus (e.g., non‐food objects) (Brockmeyer et al. 2015a; Brockmeyer et al. 2019; Beard et al. 2012; Kakoschke et al. 2017). ABMT has been reported to lower food‐related attentional bias and approach tendencies in people who binge eat (Brockmeyer et al. 2015a), and has been linked to reduced eating disorder symptoms in people with bulimia nervosa and BED (Brockmeyer et al. 2019). Transcranial direct current stimulation (tDCS), a non‐invasive form of brain stimulation, has been shown to reduce symptoms in major depressive, anxiety and substance use disorders (Moffa et al. 2020; Kekic et al. 2016). As the regulation of eating shares neural networks with general self‐regulatory processes, the dorsolateral prefrontal cortex (dlPFC) has become a primary target for intervention (Van den Eynde et al. 2013; Van den Eynde et al. 2010). Applying tDCS to the dlPFC has been reported to modulate key eating regulation processes, including reward evaluation, attention and inhibitory control, and has been shown to reduce food cravings in healthy participants and the desire to eat in overweight and obese populations (Fregni et al. 2008; Goldman et al. 2011; Montenegro et al. 2012).

ABMT combined with tDCS is a promising intervention strategy for its role in enhancing neuroplasticity to consolidate new learning (Barron et al. 2016; Heeren et al. 2015). By altering the balance of excitatory and inhibitory neurotransmitters, neuromodulation is proposed to create an environment that reinforces the avoidance behaviours learned during training (Barron et al. 2016; Heeren et al. 2015). This process likely facilitates memory reconsolidation, which is essential for sustained behavioural change (Barron et al. 2016; Heeren et al. 2015). Clinical evidence from addiction research suggests a synergistic effect of the combined approach, improving long‐term regulatory control and reducing relapse (den Uyl et al. 2017), which in the current context, would lead to a more durable reduction in food cravings and binge eating than ABMT alone.

This study investigated the feasibility of an ABMT protocol enhanced by real or sham tDCS to reduce food cravings and binge eating in individuals with SMI who were taking antipsychotic medications. Based on studies in people who experience food craving, binge eating and/or obesity (Kekic et al. 2017; Kekic et al. 2014; Jansen et al. 2013; Lowe et al. 2014; Song et al. 2018; Flynn et al. 2024; Gordon 2022), we hypothesised that participants in the combined ‘ABMT + real tDCS’ arm will show (a) decreased approach bias towards food stimuli, (b) reduced food craving and binge eating episodes compared to those in the ‘ABMT + sham tDCS’ arm, and compared to their baseline assessment.

## 2 Materials and Methods

Full methodological details of the ‘Effects of Cognitive Training and Neuromodulation on Eating Responses’ (ENTER) study have been published (Grycuk et al. 2020). In brief, this was a parallel‐group, double‐blind, two‐arm RCT, where participants were randomly allocated to receive a total of five sessions of either ‘ABMT + real tDCS’ (treatment arm) or ‘ABMT + sham tDCS’ (control arm) within 3–4 weeks, in addition to treatment as usual (TAU). To maximise study acceptability and feasibility, waiting‐list and ABMT‐only arms were not included, balancing scientific rigour with practical limitations associated with this difficult‐to‐recruit population (Beckley et al. 2025).

Outcomes were assessed at baseline (T0), post‐treatment (T1) and at a 2‐week follow‐up (T2). At the end of the study, participants in the control arm were offered the opportunity of receiving ‘ABMT + real tDCS’; however, none chose to do so.

Procedures complied with the Helsinki Declaration of 1975 (revised, 2008) and received approval from the Oxford B Research Ethics Committee (REC reference: 19/SW/0095). Written informed consent was obtained from all participants. The primary aim of the study was to assess feasibility of the intervention by assessing safety, recruitment, attendance, and retention rates, and estimating treatment effect sizes and standard deviations that might inform sample size calculations for a larger scale randomised clinical trial (RCT).

### 2.1 Participants and Setting

Eligible participants were males and females, 18–65 years old, with a current Diagnostic and Statistical Manual of Mental Disorders, 5th Edition (DSM‐5) (American Psychiatric Association 2013) diagnosis of schizophrenia, schizoaffective disorder, or psychosis unspecified, and on a stable dose of antipsychotic and/or antidepressant medication for at least 6 weeks before taking part in the study. Exclusion criteria included contraindications to tDCS (e.g., history of seizures, stroke, brain injury, frequent and severe headaches or dizziness, metal devices in the head), and significant co‐morbid medical or psychiatric conditions (e.g., substance dependence) needing treatment in their own right.

Participants were recruited between November 2019 and February 2024 (including 18‐month COVID‐19 pandemic recruitment suspension) from inpatient and community services at the South London and Maudsley NHS Foundation Trust (SLaM), as well as through online platforms, including the Institute of Psychiatry, Psychology and Neuroscience (IoPPN) website, *Call for Participants*, *MQ Mental Health Research website*, and social media channels from the Centre for Research in Eating and Weight Disorders at King's College London (CREW‐KCL). The study was conducted at the Institute of Psychiatry, Psychology and Neuroscience at KCL and at inpatient and community services at SLaM.

### 2.2 Sample Size, Randomisation and Blinding

As a feasibility trial, this study did not require a priori sample calculation. A target sample size of 30 participants was selected, consistent with the recommended range of 24–50 participants for feasibility trials: this is sufficient to estimate effect sizes of continuous variables, which can inform sample size calculations for future large‐scale clinical trials (Lancaster et al. 2004; Julious 2005).

Randomisation was conducted using the *Sealed Envelope* online randomisation tool (Sealed Envelope Ltd. 2019) by an independent researcher from KCL who was not part of the ENTER team. Participants were allocated in a 1:1 ratio to one of the two trial arms following completion of the baseline assessment.

Blinding of participants and researchers to treatment allocation was ensured through the trial's parallel design. The tDCS devices incorporated a built‐in blinding feature, delivering 30 s of real stimulation (vs. 20 min for the active group) to replicate skin sensations usually reported with real stimulation. To assess blinding success, participants were asked to guess their treatment allocation in their post‐assessment session.

### 2.3 Intervention

Participants received a total of five sessions of ABMT with either real or sham tDCS within 3–4 weeks, in addition to TAU (Figure 1). Each session lasted 30–40 min, inclusive of preparation time, 20 min of simultaneous tDCS and ABMT, and the administration of within‐session assessments.

**FIGURE 1:**
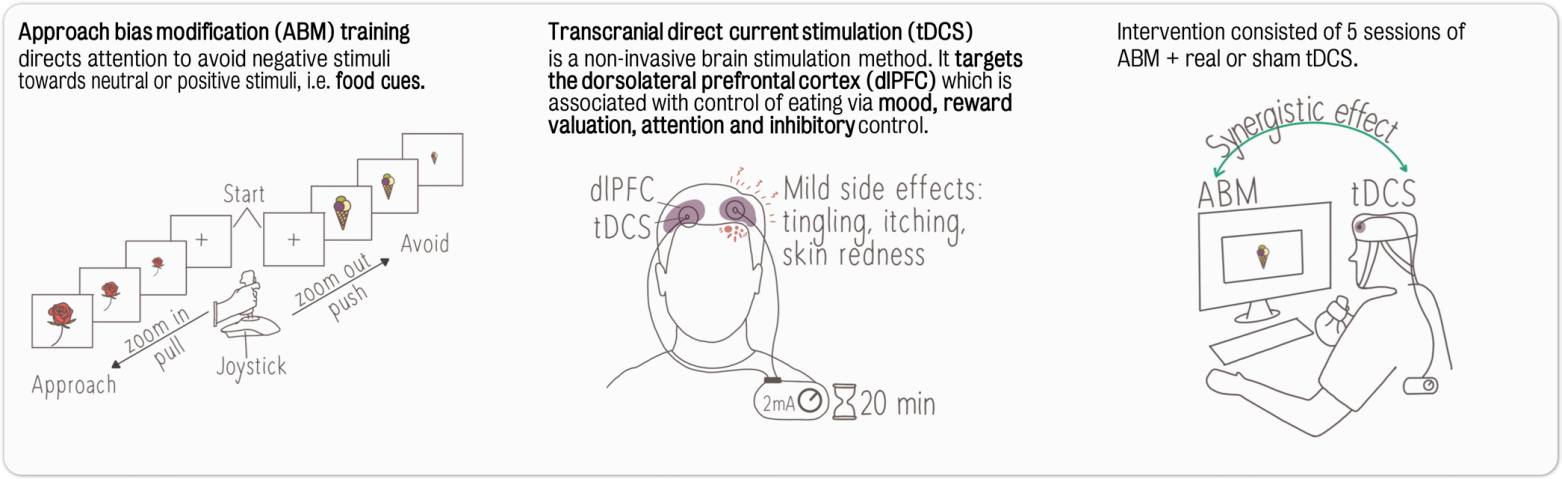
Participants received five sessions of approach bias modification (ABM) training and real or sham transcranial direct current stimulation (tDCS).

The cognitive training (ABMT) was an adapted version of the Food Approach/Avoidance Task (F‐AAT) (Brockmeyer et al. 2016; Blechert et al. 2014). This task employs an implicit learning paradigm, requiring participants to “push” a joystick (i.e., avoid) in response to all food pictures and “pull” the joystick (i.e., approach) in response to pictures of a non‐edible object. For more details on the task, see protocol paper (Grycuk et al. 2020).

TDCS (real and sham) was delivered at a constant current of 2 mA (with a 10‐s fade in/out) using two 25 cm^2^ surface sponge electrodes soaked in sterile saline solution (0.9% sodium chloride). Until March 2020, the neuroConn DC‐Stimulator device was utilised. Following the onset of the COVID‐19 pandemic, a self‐administered HDC Kit with MindCap by Newronika was employed (further details in ‘Changes to Protocol’ section). The stimulation site was determined using the International 10–20 system, with a bilateral montage: anode (a positively charged electrode) placed over the right dlPFC (F4 location) and the cathode (a negatively charged electrode) over the left dlPFC (F3 location) (Jasper 1958).

In the real tDCS group, participants received continuous stimulation for 20 min, while in the sham tDCS group, stimulation was applied for 30 s before being automatically deactivated. The study followed the recommended procedures and parameters to guarantee participants' safety, and tDCS was delivered by trained researchers (Brunoni et al. 2012). Although tDCS is well‐tolerated by patients, with minimal and mild side effects (Poreisz et al. 2007; Brunoni et al. 2011), the presence of adverse effects was recorded for each participant, and, as a safety measure, blood pressure and pulse were measured before and after each treatment.

There is no consensus on standardised treatment duration and frequency for ABMT and tDCS (Jones and Sharpe 2017; Menne‐Lothmann et al. 2014; Beard et al. 2012; Sabé et al. 2024). There is evidence from alcohol research of a maximum cumulative effect of ABMT at six sessions (Eberl et al. 2014), but tDCS has demonstrated potential to reduce food cravings after a single session (Burgess et al. 2016). Some evidence suggests that multiple sessions are superior for inducing long‐term therapeutic effects (Kekic et al. 2016). Therefore, the ENTER study adopted a patient‐centred approach, offering flexibility while ensuring an ABMT dose at the threshold of maximum effect.

### 2.4 Procedure

Full procedures and measures‐by‐assessment, consistent with the SPIRIT Statement (Chan et al. 2013), are detailed in the protocol paper (Grycuk et al. 2020). Eligibility of potential participants, whether referred or self‐referred, was confirmed in a screening call, and written informed consent was obtained from all participants prior to the baseline assessment.

Apart from the tDCS stimulation, procedures were identical between the two groups. During the baseline assessment (T0), participants completed a battery of questionnaires and experimental tasks assessing eating behaviours and mood, and approach bias towards food cues (described below). Following the baseline assessment, participants were randomly allocated to the treatment group ‘ABMT + real tDCS’ or control group ‘ABMT + sham tDCS’. Post‐treatment assessment (T1) was conducted after the fifth treatment session and included the same set of questionnaires and experimental tasks as at baseline. A follow‐up assessment (T2) was completed 2 weeks later, focusing on mood, food cravings, and binge eating behaviours.

### 2.5 Outcomes

Trial feasibility was assessed via a range of outcomes, including recruitment, attendance and retention rates (primary feasibility outcomes), blinding success and acceptability, based on previous feasibility studies by our groups (Flynn et al. 2024; Brockmeyer et al. 2015b). A recruitment rate of 2.5 participants/month was calculated based on the study's expected 12‐month duration and a target sample size of 30 participants. According to the study protocol, progression for a future large‐scale RCT would be based on the number of patients recruited, the proportion retained, and completing the intervention, the acceptability and tolerability of the tDCS and the effect sizes of treatment outcomes.

Blinding success was defined as participants guessing their group allocation (real or sham) at a rate comparable with chance (i.e., < 50% accuracy). Intervention acceptability was assessed using the following criteria: (a) visual analogue scale (VAS) scores on the levels of tension, stress and anxiety measured before and after each treatment session; (b) side‐effect frequency and severity were recorded before and after each treatment session; (c) at the end of the study, participants were asked whether they would consider participating in a therapeutic tDCS trial if available; and (d) the number of recruited participants. An endorsement rate of 75% or higher would indicate the intervention is acceptable. Treatment tolerability was assessed after each treatment session using VAS to measure discomfort levels. The intervention would be considered well‐tolerated if the mean VAS score across the group remained ≤ 40 points, corresponding to mild discomfort.

### 2.6 Clinical Outcomes

Clinical outcomes assessed were changes in food cravings, and the number of objective binge‐eating episodes from T0 to T1 and T2. Other outcomes included changes in body mass index (BMI), general psychopathology (schizophrenia symptoms, impulsivity, cognition, and mood), approach bias towards food, and eating disorder symptoms.

Food craving traits were assessed at T0, T1 and T2 using the Food Cravings Questionnaire‐Trait‐Reduced (FCQ‐T‐r) (Meule et al. 2014). Objective binge‐eating episodes (defined as the consumption of a large amount of food within a defined period accompanied by a sense of loss of control over eating), and eating disorder symptoms during the past 28 days were assessed using the Eating Disorder Examination Questionnaire (EDE‐Q) (Fairburn and Beglin 2008). BMI was calculated as body weight (measured at in‐person assessments) divided by self‐reported squared height.

At T0 and T1, schizophrenia symptoms were assessed using the Simplified Negative and Positive Symptoms Interview (SNAPSI) (Østergaard et al. 2017) and the Positive and Negative Syndrome Scale (PANSS‐6) (Østergaard et al. 2016). Impulsivity was measured with the Barratt Impulsiveness Scale (BIS‐11) (Burgess et al. 2016) and cognition impairments were assessed using the Montreal Cognitive Assessment (MoCA) (Nasreddine et al. 2005). Mood was assessed at T0, T1, and T2 using the Depression, Anxiety and Stress Scale (DASS‐21) (Lovibond and Lovibond 1995). Approach bias towards high‐calorie food items was measured using two computer‐based tasks at baseline and post‐treatment, including a physical Food Approach‐Avoidance Task (F‐AAT) (Brockmeyer et al. 2015a) and the Stimulus–Response Compatibility Task (SRCT) (De Houwer et al. 2001). The F‐AAT measured participants' reaction times when pulling or pushing a joystick in response to visual cues of high‐calorie food versus non‐edible objects. Positive values indicate an approach bias to high‐calorie food (pulling food pictures faster than pushing them), whereas negative values indicate an avoidance bias to high‐calorie food (pulling food pictures slower than pushing them) (Brockmeyer et al. 2015a). The SRCT measured participants' reaction times when pressing keys to move a character on the screen towards or away from images of food versus non‐edible objects. Faster movements towards food pictures indicate an approach bias, while faster movements away from food pictures indicate an avoidance bias (Blechert et al. 2014).

### 2.7 COVID‐Related Changes to Protocol

Changes to the published protocol were made due to the COVID‐19 pandemic, including implementation of risk assessments before each session, and adherence to governmental social distancing and mitigation guidelines (hands disinfection, wearing face masks, and maintaining a 2‐m distance). The neuroConn DC‐Stimulator was replaced by the HDC Kit with MindCap by Newronika, designed for self‐administration. Participants (*n* = 13, 46.4%) were trained via manufacturer instructional videos and printed manuals, with all sessions conducted under researcher supervision to ensure correct electrode placement and protocol adherence. The food challenge task (Kekic et al. 2017) examining cue‐induced food craving (Food Cravings Questionnaire‐State) and the Bogus Taste Test, which measured actual food consumption, were omitted from baseline and post‐treatment assessments. The original 12‐month trial, starting from the first participant, was extended to 31 months because of an 18‐month delay. This resulted in 13 months of active trial duration.

### 2.8 Data Analyses

Analyses were performed on SPSS software adopting intention‐to‐treat principles. Descriptive statistical analyses and graphical methods were applied to determine the quality, completeness and variability of the outcome measures. Variables are presented as means (standard deviations) or frequencies (%), and a significance level of 0.05 was adopted. Group differences were investigated using independent t‐test for normally distributed continuous variables, Mann–Whitney test for non‐normally distributed continuous variables, and Chi‐Squared tests for categorical variables. The size of the treatment effect (Cohen's *d*) on clinical outcomes was calculated for the differences between T0 and T1, and T0 and T2 (small effect: *d* ≥ 0.2, moderate effect: *d* ≥ 0.5, and large effect: *d* ≥ 0.8). Between group and within‐groups differences were assessed using independent and paired *t*‐tests, respectively. The Missing Completely at Random (MCaR) test indicated the data were missing completely at random (*χ* ^2^ = 38.8; *p* = 0.6), which was handled by pairwise deletion. Per‐protocol analyses were also performed and are available in Supporting Informations S3.

## 3 Results

### 3.1 Feasibility Outcomes

#### 3.1.1 Participant Flow, Recruitment, Retention and Acceptability

A CONSORT diagram of participant flow is presented (Figure 2). Baseline assessments were offered to 42 individuals but only completed by 28. Non‐completion was attributed to reasons, including inability to contact, lack of interest, unavailability, a positive COVID‐19 test, refusal to wear a face mask, missed appointments, and changes in medication. A total of 28 people were randomised to receive either ‘ABMT + real tDCS’ (*n* = 14) or ‘ABMT + sham tDCS’ (*n* = 13). One participant in the latter group was found ineligible and excluded due to an inability to comprehend the study and complete the questionnaires. Recruitment was extended to 31 months to address challenges posed by the COVID‐19 pandemic, ultimately achieving 93% of the target sample size, with a recruitment rate of 0.9 out of ~2.5 participants per month.

**FIGURE 2:**
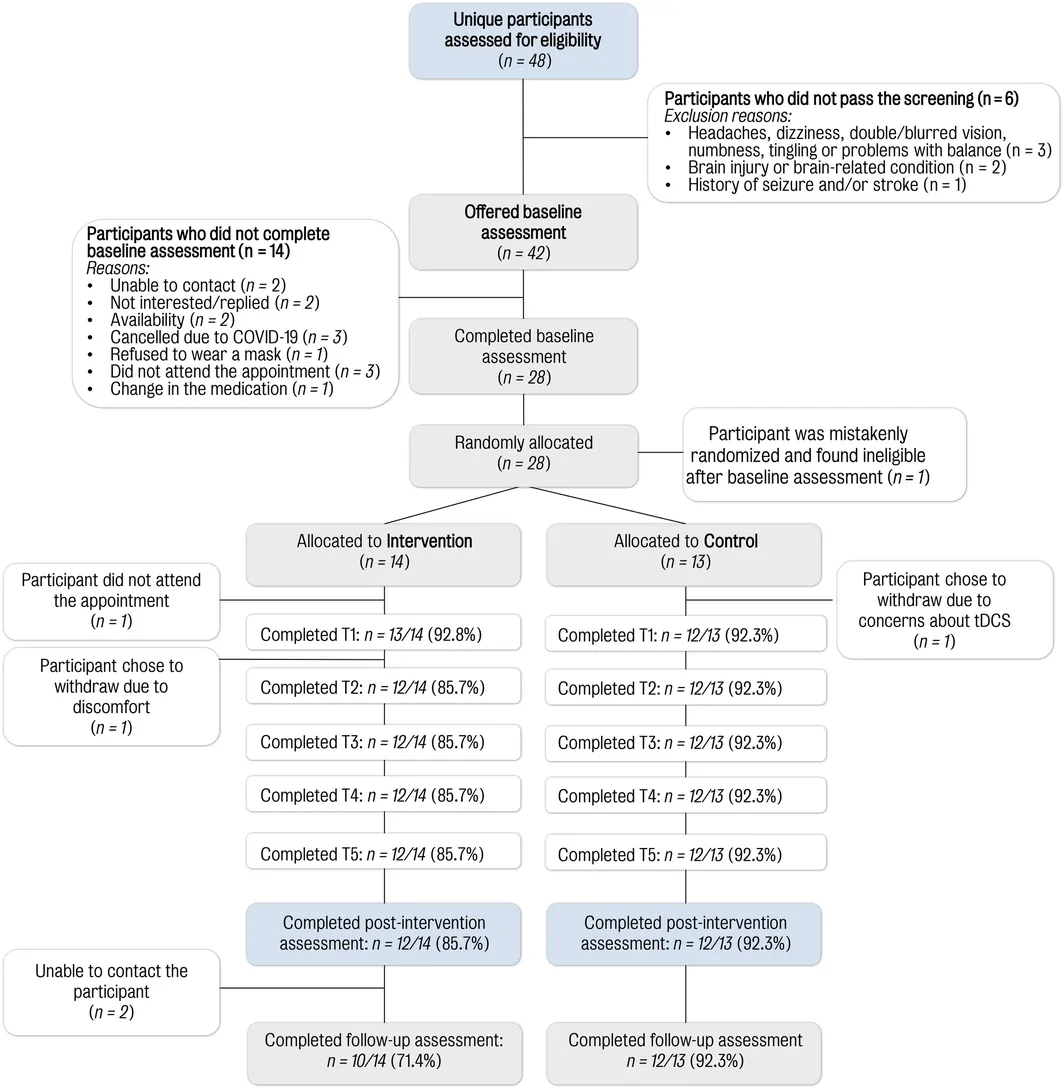
CONSORT diagram of participant flow.

In the ‘ABMT + real tDCS’ group, one participant did not attend the treatment appointments, and another withdrew during the first session due to discomfort. In the ‘ABMT + sham tDCS’ group, one participant withdrew after randomisation due to concerns about tDCS. The remaining 24 participants (85.7%) completed treatment and post‐assessment sessions (ABMT + real tDCS: *n* = 12, 85.7% and ABMT + sham tDCS: *n* = 12, 92.3%). The T2 follow‐up assessment was completed by 22 participants (78.6%), 12 from the ‘ABMT + sham tDCS’ arm (92.3%) and 10 from the ‘ABMT + real tDCS’ arm (71.4%). No significant differences in retention rates were found between treatment and control arms. Blinding accuracy for the whole group was below chance at 46.4% and did not differ significantly between groups (ABMT + real tDCS: *n* = 7, 58.3% and ABMT + sham tDCS: *n* = 6, 50.0%; *p* = 0.5). Researchers administering tDCS were also blinded, but their blinding was compromised by more frequent failures caused by low impedance when using the real tDCS device.

There were no significant differences between trial arms in the frequency, number, and incidence of side‐effects events (ABMT + real tDCS: 78.7%, ABMT + sham tDCS: 68.3%; *χ* ^2^ = 1.67; *p* = 0.197). Commonly reported side effects (ABMT + real tDCS: 5%–24.6%; ABMT + sham tDCS: 5%–17%) included itching, heating/warmth, stinging, tingling, headache or pain. Participants in the ‘ABMT + real tDCS’ arm additionally reported light‐headedness, skin redness, eye dryness, burning sensations and discomfort, while one in the sham arm reported tiredness (Supporting Information S2).

The endorsement rate for participating in a therapeutic trial of ABMT + tDCS for the entire group was 75% (ABMT + real tDCS: 78.6% ABMT + sham tDCS: 76.9%). Regarding tolerability, both arms reported mild discomfort during stimulation (ABMT + real tDCS: *M* = 21.0, SD = 13; ABMT + sham tDCS: *M* = 20.0, SD = 14; *p* = 0.8), which appeared to decrease as treatment progressed. For more details on intervention acceptability and tolerability see Supporting Informations S1 and S2.

#### 3.1.2 Baseline Characteristics

Participants' demographics and baseline clinical characteristics are presented in Table 1. No significant differences between arms were observed at T0. Participants (63% females) had a mean age of 46.8 years (SD = 9.3), a mean illness duration of 12.9 years (SD = 8.8), mild schizophrenia symptoms (PANSS‐6: *M* = 12.7, SD = 4.9), and mild cognitive impairment (MoCA: *M* = 23.5, SD = 3.5). Of the total sample, 81.5% reported using a second‐generation antipsychotic and 18.5% reported using a first‐generation drug, with no significant group differences. Mean BMI was 32.5 kg/m^2^ (SD = 6.4), mean frequency of binge eating episodes was 6.3 over 28 days (SD = 10.7) and in the FCQ‐T, the group had a mean of 52.3 points/90 (SD = 18.9).

**TABLE 1:** Baseline characteristics.

		ABMT + Real tDCS		ABMT + Sham tDCS	
		(*n* = 14)		(*n* = 13)	
		Mean or *n*	SD or %	Mean or *n*	SD or %
Age (years)		47.4	9.8	46.2	9.3
Female		8	57.1	9	69.2
Right‐handed		12	85.7	11	84.6
Occupation	Full or part‐time	4	28.6	4	30.8
	Voluntary	1	7.1	2	15.4
	Unemployed	9	64.3	7	53.8
Marital status	Single	12	85.7	11	84.6
	Married	1	7.1	1	7.7
Education	GCSE	4	28.6	7	58.3
	AS and above	10	71.4	4	33.3
Ethnicity	Caucasian	7	50.0	5	38.5
	Black	4	28.6	4	30.8
	Asian	0	0.0	1	7.7
	Mixed	3	21.4	3	23.1
BMI (kg/m^2^)		30.2	5.7	34.9	6.5
Illness duration (years)		14.0	9.8	11.7	8.0
PANSS‐6 total (range 7–42)		12.6	5.5	12.8	4.6
MoCA total (normal ≥ 26)		22.6	4.1	24.4	2.8
DASS‐21 total		32.6	25.9	48.3	27.2
DASS‐21 depression		9.2	10.5	16.6	11.6
DASS‐21 stress		11.6	8.7	18.0	9.8
DASS‐21 anxiety		11.8	9.9	13.7	9.5
BIS‐11 total		132.7	20.3	145.5	14.3
EDE‐Q global		2.3	1.3	2.7	1.1
EDE‐Q Binge episodes		9.2	13.5	3.3	5.5
FCQ‐T		53.1	20.0	51.5	18.5
F‐AAT approach bias (ms)		27.4	73.7	10.9	80.2
SRCT approach bias (ms)		198.6	365.1	−59.4	406.3
SRCT avoidance bias (ms)		7.8	869.2	30.3	287.7
SRCT approach‐avoidance bias (ms)		409.2*	528.1	−89.7	503.9
First/second‐generation antipsychotic		3/11	21.4/78.6	2/11	15.4/84.6
Clozapine		4	28.6	1	7.7
Smoking (yes)		7	53.8	5	38.5
Alcohol use (yes)		5	41.7	3	23.1
Drug use (yes)		1	8.3	1	8.3

Abbreviations: ABMT, approach bias modification training; BMI, body mass index; DASS‐21, Depression, Anxiety and Stress Scale—21 items; BIS‐11, Barratt Impulsiveness Scale; EDE‐Q, Eating Disorder Examination Questionnaire; F‐AAT, Food Approach/Avoidance Task; FCQ‐Tr, Food Craving Questionnaire‐Trait reduced version; MoCA, Montreal Cognitive Assessment; n, number of observations, PANSS‐6, Positive And Negative Syndrome Scale; SD, standard deviation; SRCT, Stimulus–Response Compatibility Task; tDCS, transcranial direct current stimulation.

* Significant difference at the *p* < 0.05 level in Independent T/Mann–Whitney and Chi‐squared tests.

Average binge eating episodes were not significantly different at baseline between the two arms (*χ* ^2^ = 1.25, *p* = 0.535), with 10 or more episodes reported by 42.9% in the real arm and 23.1% in the sham arm, between 1 and 3 episodes reported by 14.3% in the real arm and 15.4% in the sham arm, and no episode reported by 42.9% in the real arm and 61.5% in the sham arm.

On the SRCT task, participants in the ‘ABMT + sham tDCS’ arm showed an average baseline avoidance bias towards food, while the ‘ABMT + real tDCS’ arm showed an average approach bias to food (*p* < 0.05). F‐AAT measured positive approach bias towards food in both arms. A food‐specific approach bias was observed in 64.3% (*n* = 9) of the ‘ABMT + real tDCS’ arm and 45.5% (*n* = 5) of the ‘ABMT + sham tDCS’ arm (*χ* ^2^ = 1.8, *p* = 0.2). A food‐specific avoidance bias was observed in 35.7% (*n* = 5) ‘ABMT + real tDCS’ arm and 54.5% (*n* = 6) of the ‘ABMT + sham tDCS’ arm (*χ* ^2^ = 0.3, *p* = 0.6).

### 3.2 Clinical Outcomes

Mean change scores and effect sizes of clinical variables (T1–T0) and (T2–T0) between groups are presented for between‐group and within‐groups in Table 2.

**TABLE 2:** Mean change scores for clinical outcome measures from post‐treatment (T1) to baseline (T0) and from follow‐up (T2) to baseline (T0), including within‐groups and between‐group effect sizes (Cohen's *d* with 95% confidence intervals).

	ABMT + Real t‐DCS							ABMT + Sham t‐DCS							Between‐groups effects			
	*n*	Mean	SD	Cohen's *d*	Low CI	Upper CI	*p*	*n*	Mean	SD	Cohen's *d*	Low CI	Upper CI	*p*	Cohen's *d*	Low CI	Upper CI	*p*
Change from T1–T0																		
PANSS‐6 total	10	0.0	1.2	0.0	−0.6	0.6	1.000	10	−0.6	1.0	−0.6	−1.3	0.1	0.081	0.5	−0.4	1.4	0.245
BMI (kg/m^2^)	10	−0.1	0.8	−0.1	−0.7	0.5	0.718	10	−0.1	0.3	−0.4	−1.1	0.2	0.221	0.0	−0.8	0.9	0.951
DASS−21 total	10	−0.8	17.2	0.0	−0.7	0.6	0.887	10	−2.4	21.1	−0.1	−0.7	0.5	0.728	0.1	−0.8	1.0	0.855
DASS−21 depression	10	−3.0	6.9	−0.4	−1.1	0.2	0.201	10	−1.2	7.6	−0.2	−0.8	0.5	0.630	−0.2	−1.1	0.6	0.586
DASS−21 stress	10	0.8	5.8	0.1	−0.5	0.8	0.674	10	−2.0	7.0	−0.3	−0.9	0.4	0.389	0.4	−0.5	1.3	0.344
DASS−21 anxiety	10	1.4	7.2	0.2	−0.4	0.8	0.556	10	0.4	10.1	0.0	−0.6	0.7	0.904	0.1	−0.8	1.0	0.803
BIS−11 total	10	−0.6	6.7	−0.1	−0.7	0.5	0.786	10	−1.4	15.8	−0.1	−0.7	0.5	0.786	0.1	−0.8	0.9	0.884
EDE−Q global	10	−0.2	0.7	−0.3	−1.0	0.3	0.320	10	0.0	0.7	0.0	−0.7	0.6	0.905	−0.3	−1.2	0.6	0.514
EDE−Q Binge episodes	10	−6.3	14.7	−0.4	−1.1	0.2	0.208	10	0.0	2.2	0.0	−0.6	0.6	1.000	−0.6	−1.5	0.3	0.211
FCQ−T	10	−5.2	18.7	−0.3	−0.9	0.4	0.402	10	−4.0	14.6	−0.3	−0.9	0.4	0.410	−0.1	−0.9	0.8	0.875
F−AAT—approach bias to food	10	−28.5	39.4	−0.7*	−1.4	0.0	0.048	10	−28.3	92.3	−0.3	−0.9	0.3	0.358	0.0	−0.9	0.9	0.996
SRCT—approach bias to food	10	−307.9	455.3	−0.7	−1.4	0.0	0.061	10	−176.9	481.4	−0.4	−1.0	0.3	0.275	−0.3	−1.2	0.6	0.540
SRCT—avoidance bias to food	10	290.6	351.0	0.8*	0.1	1.5	0.028	10	−39.6	344.6	−0.1	−0.7	0.5	0.725	0.9*	0.0	1.9	0.048
SRCT—approach−avoidance bias to food	10	−598.5	756.0	−0.8*	−1.5	−0.1	0.034	10	−137.4	547.1	−0.3	−0.9	0.4	0.448	−0.7	−1.6	0.2	0.136
Change from T2–T0																		
DASS−21 total	10	−1.7	19.4	−0.1	−0.7	0.5	0.787	11	−15.5	27.2	−0.6	−1.2	0.1	0.087	0.6	−0.3	1.5	0.199
DASS−21 depression	10	0.2	5.9	0.0	−0.6	0.7	0.917	11	−4.1	11.1	−0.4	−1.0	0.2	0.244	0.5	−0.4	1.3	0.275
DASS−21 stress	10	2.4	7.9	0.3	−0.3	0.9	0.364	11	−4.4	12.6	−0.3	−0.9	0.3	0.277	0.6	−0.3	1.5	0.162
DASS−21 anxiety	10	−4.3	9.3	−0.5	−1.1	0.2	0.177	11	−5.3	9.0	−0.6	−1.2	0.1	0.081	0.1	−0.8	1.0	0.810
EDE−Q Binge episodes	10	−6.7	9.3	−0.7*	−1.4	0.0	0.049	11	−2.4	5.1	−0.5	−1.1	0.2	0.157	−0.6	−1.5	0.3	0.197
FCQ−T	10	−0.8	9.6	−0.1	−0.7	0.5	0.797	11	−6.9	13.0	−0.5	−1.2	0.1	0.108	0.5	−0.3	1.4	0.238

*Note:* Post‐assessment/ follow‐up scores minus baseline scores.

Abbreviations: ABMT, approach bias modification training; BIS‐11, Barratt Impulsiveness Scale; BMI, body mass index; DASS‐21, Depression, Anxiety and Stress Scale—21 items; EDE‐Q, Eating Disorder Examination Questionnaire; F‐AAT, Food Approach/Avoidance Task; FCQ‐Tr, Food Cravings Questionnaire‐Trait reduced version; MoCA, Montreal Cognitive Assessment; *n*, number of observations, PANSS‐6, Positive And Negative Syndrome Scale; SD, standard deviation; SRCT, Stimulus–Response Compatibility Task; tDCS, transcranial direct current stimulation.

* Significant difference at the *p* < 0.05 level for CIs and in paired *t*‐tests.

#### 3.2.1 Clinical Outcomes Related to Eating Behaviours

Change scores indicated reductions in binge eating episodes and food craving traits in both arms. Medium between‐group effect sizes in binge eating change scores (T2–T0) favoured ‘ABMT + real tDCS’ (real: *M* = −6.7, SD = 9.3; sham: *M* = −2.4, SD = 5.1; *d* = −0.6, 95% CI −1.5 to 0.3), while changes in food craving scores favoured ‘ABMT + sham tDCS’ (real: *M* = −0.8, SD = 9.6; sham: *M* = −6.9, SD = 13.0; *d* = 0.5, 95% CI −0.4 to 1.4). Within‐groups analyses in the ‘ABMT + real tDCS’ arm showed significant reductions in binge eating change scores between T2 and T0 (*p* = 0.049), with a moderate effect size (*d* = −0.7), while no significant changes were observed in the ‘ABMT + sham tDCS’ arm (*p* = 0.157; *d* = −0.5) (Figure 3). This result remained consistent in per‐protocol analyses.

**FIGURE 3:**
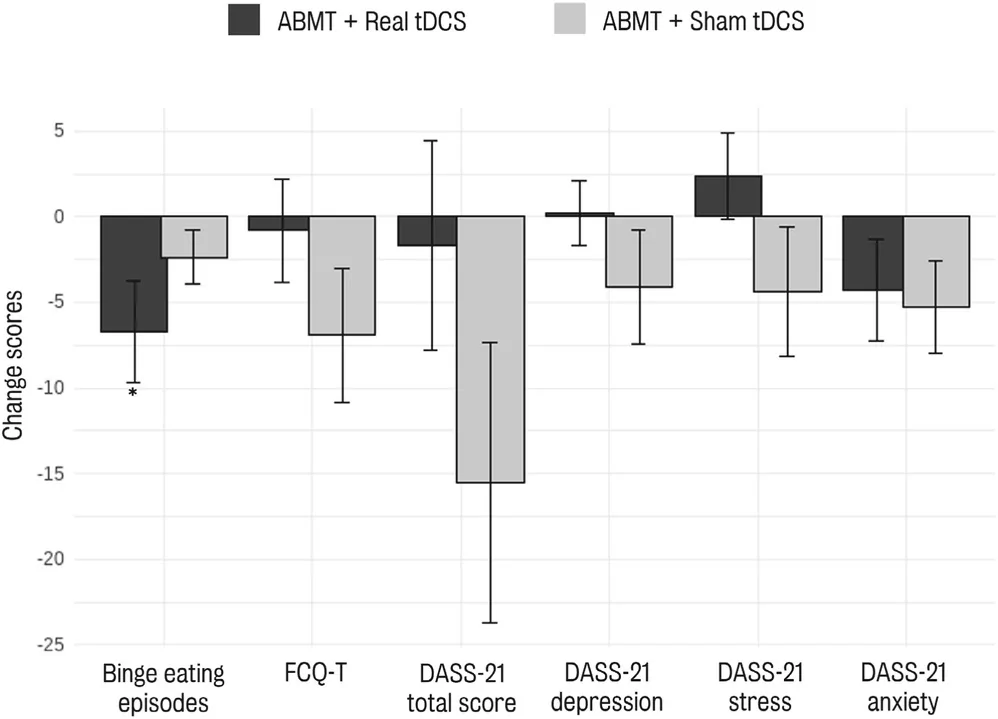
Change scores of clinical outcomes at 2‐week follow‐up (T2). Follow‐up score minus baseline score. *Statistical difference (*p* < 0.05) in paired t‐tests between T0 and T2. ABMT, approach bias modification training; BMI, body mass index; DASS‐21, Depression, Anxiety and Stress Scale—21 items; EDE‐Q, Eating Disorders Examination Questionnaire; FCQ‐Tr, Food Craving Questionnaire‐Trait reduced version; tDCS, transcranial direct current stimulation.

After the intervention, both groups showed negative change scores in F‐AAT approach bias, with a very small effect size (*d* < 0.1) favouring the ‘ABMT + real tDCS’ arm. Changes in SRCT approach bias to food, avoidance bias to food and approach‐avoidance bias to food favoured the ‘ABMT + real tDCS’ arm, with small to large effect sizes, respectively (*d* = −0.3 95% CI −1.2 to 0.6, *d* = 0.9 95% CI 0.0–1.9 and *d* = −0.7 95% CI −1.6 to 0.2). In within‐group analyses, only the ‘ABMT + real tDCS’ arm showed significant reductions in F‐AAT approach bias to food (*d* = −0.7 95% CI −1.4 to −0.1), and in SRCT approach‐avoidance bias to food (*d* = −0.8 95% CI −1.5 to −0.1). SRCT avoidance bias to food increased with significant large effect sizes (*d* = 0.8 95% CI 0.1–1.5). Between T0 and T1, BMI varied between −0.096 kg/m² in the real arm and −0.113 kg/m² in the sham arm, with negligible effect size (*d* = 0.0, 95% CI –0.9 to 0.9) (Figure 4). In per‐protocol analyses, only the SRCT approach‐avoidance bias to food reduction remained consistent.

**FIGURE 4:**
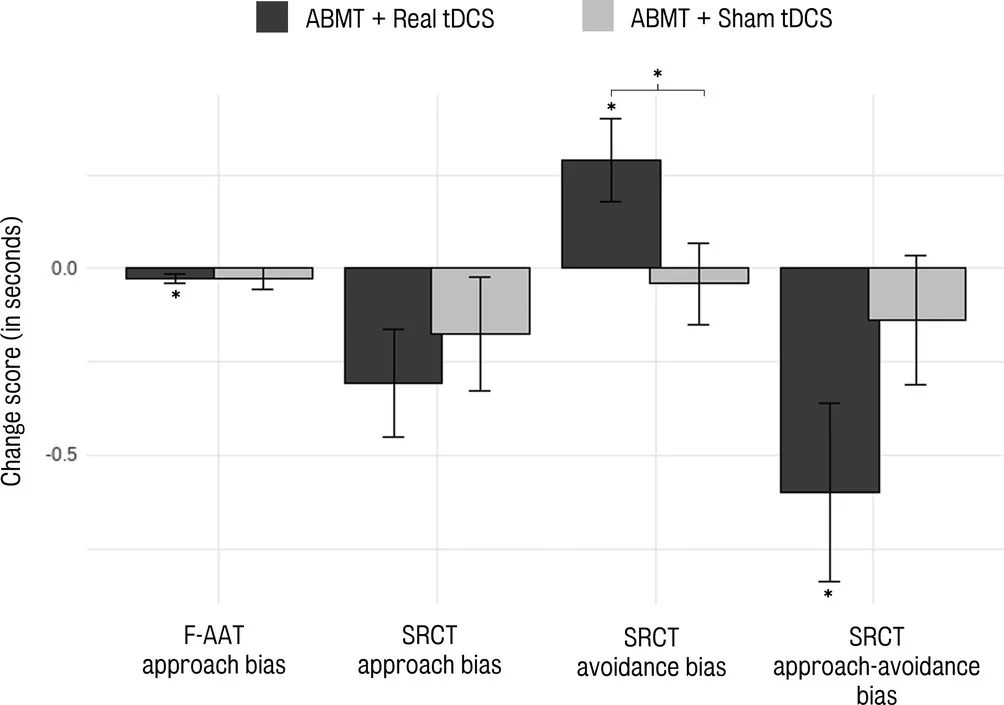
Change scores of clinical outcomes at post‐treatment assessment (T1). Post‐assessment score minus baseline score. *Statistical difference (*p* < 0.05) in paired/independent t‐tests between T0 and T1. ABMT, approach bias modification training; F‐AAT, Food Approach/Avoidance Task; SRCT, Stimulus–Response Compatibility Task; tDCS, transcranial direct current stimulation.

#### 3.2.2 Other Clinical Outcomes

Change scores of psychopathological symptoms between T0 and T1 showed small to moderate effects. Symptoms of schizophrenia remained similar in the ‘ABMT + real tDCS’ arm and slightly decreased in the ‘ABMT + sham tDCS’ arm (*d* = 0.6, 95% CI –0.4 to 1.4). Impulsivity, measured by the BIS‐11 total score, slightly favoured the ‘ABMT + sham tDCS’ arm (*d* = 0.1, 95% CI –0.8 to 0.9). The EDE‐Q global score slightly favoured the ‘ABMT + real tDCS’ arm (*d* = −0.3, 95% CI –1.2 to 0.6). In per‐protocol analyses, PANSS‐6 reduction favoured the sham group with a large effect‐size (*d* = 1.0 95% CI 0.0–1.8). Due to COVID‐19 impact, MoCA was only applied to part of the sample. These results were analysed in per‐protocol analyses only, showing that a small effect size favoured an increase in MoCA change scores for the ‘ABMT + sham tDCS’ arm (*d* = −0.3, 95% CI –1.3 to 0.5).

The average of total DASS change score was negative in both arms at both timepoints (T1 and T2), with a moderate effect size and favoured the ‘ABMT + sham tDCS’ arm at T2 (*d* = 0.6, 95% CI –0.3 to 1.4). Depression symptoms decreased in both arms from T0 and T1, however, this decrease was only maintained at T2 in the ‘ABMT + sham tDCS’ arm, with a moderate effect size (*d* = 0.5, 95% CI –0.4 to 1.4). At both time points, stress symptoms increased in the ‘ABMT + real tDCS’ arm and decreased in the ‘ABMT + sham tDCS’ arm, with moderate effect sizes at T2 (*d* = 0.6, 95% CI –0.2 to 1.5). Anxiety change scores were positive for both arms but turned to negative at T2 with small effect size favouring the ‘ABMT + sham tDCS’ arm (*d* = 0.1 95% CI −0.7 to 0.9). In per protocol analyses, anxiety results were consistent, and we observed a significant large effect size of the sham arm (*d* = −0.6, 95% CI −1.2 to −0.0), as well a large effect size on stress favouring the sham arm (*d* = 0.9 95% CI 0.0–1.8).

## 4 Discussion

This study assessed the feasibility of an RCT combining ABMT with tDCS to reduce food cravings and binge eating in people with SMI and taking antipsychotics. Our data indicate that, while efforts are needed to improve recruitment, the intervention protocol is feasible and acceptable, with high retention rates for post‐treatment assessment and follow‐up, alongside successful blinding. These results support further consideration of a confirmatory trial. Preliminary efficacy outcomes suggest that participants who received ‘ABMT + real tDCS’ had reductions in binge eating episodes, and this was accompanied by a reduction in approach bias to food and an increase in avoidance bias to food (a potential effect of ABMT).

### 4.1 Feasibility Outcomes

The study recruited 28 participants, achieving 93% of the target sample size (*N* = 30). Consistent with previous feasibility RCTs by our group combining tDCS with different cognitive trainings, recruitment fell short by one or two participants post‐randomisation (Flynn et al. 2024; Gordon 2022). The COVID‐19 pandemic led to changes in the study procedures, and to lower and more challenging recruitment. However, ENTER was able to demonstrate robust feasibility. It had a completion rate of 85% and a retention rate of > 75%. This was comparable to feasibility studies using tDCS with cognitive training in binge eating disorder (BED) (Flynn et al. 2024; Gordon 2022), as well as ABMT trials for alcohol‐dependence (Manning et al. 2016) and tDCS trials for auditory hallucinations in schizophrenia (Mellin et al. 2018). Feasibility trials reaching a recruitment rate > 90%, completion rate ≥ 80% and a retention‐to‐follow‐up rate ≥ 75% support progression to a full trial with relevant adjustments (Grycuk et al. 2025).

The ENTER study demonstrates the need for a recruitment strategy tailored to individuals with SMI. Motivations for trial enrolment were the potential for improved eating behaviours, other treatment benefits and monetary compensation. Participants had concerns regarding tDCS safety, particularly its perceived association with electroconvulsive therapy and, somewhat surprisingly, lobotomy (Grycuk et al. 2021). They also had some misconceptions about enhanced concentration or of replacing treatment as usual (Grycuk et al. 2021). This could be related to the mild cognitive impairment observed in our sample and previously reported in individuals with schizophrenia (Fioravanti et al. 2012). To address these issues, a recruitment approach should: (i) provide clear, simple, and repeated information and messaging, (ii) simplify participation processes, (iii) provide educational materials to alleviate fears and misconceptions around brain stimulation, (iv) offer a trial session of tDCS, and (v) leverage established relationships of participants within psychosis services (Grycuk et al. 2021).

Blinding was successful, with participants guessing their allocation at chance levels (~ 50%). The real tDCS group showed 8% higher blinding accuracy than the sham group, although this difference was not statistically significant. In addition, researchers in our study could sometimes suspect the treatment condition due to device errors, such as impedance failure. Other studies have raised concerns regarding tDCS blinding, including the effectiveness of a “fade in, fade out” sham tDCS strategy (Turner et al. 2021; Fonteneau et al. 2019), and redness under the electrodes in the active stimulation condition (O'Connell et al. 2012). Skin redness caused by increased blood flow (erythema) is the most common side effect (observed in 80% of cases). It is usually not perceived by participants but could compromise double‐blinded trials. Future protocols should improve placebo control by exploring options such as including online probe questions during stimulation, stimulating an unrelated cortical site, using anodal versus cathodal protocols, applying anaesthetic on the stimulation site in both conditions, or employing methods that mimic active stimulation without neural effects (e.g., ActiSham (Turner et al. 2021)).

No adverse events, or serious adverse reactions were reported. Over 75% of participants endorsed the treatment, and more than 85% completed it, with a slightly higher completion rate in the sham group (92.3%) than in the real tDCS arm (85.7%). One participant in the real condition withdrew due to discomfort during the first session. Our results accord with previous studies (Flynn et al. 2024; Gordon 2022), in which ~30% of participants receiving non‐invasive brain stimulation reported side effects such as tingling, paraesthesia, skin redness, and discomfort at the application site (Brunoni et al. 2011). Given that 6% of the sample were women, it is of note that women report more severe sensations from tDCS than men (Workman et al. 2021), which could have influenced tolerability. Our findings support the feasibility of tDCS as a safe and acceptable intervention for individuals taking antipsychotic medication, as side effects were manageable and did not significantly impact adherence. As previously suggested, a tDCS taster session in future clinical trials could help participants familiarise themselves with the procedure and reduce withdrawals due to discomfort. Stratifying randomisation by sex may help assess potential differences in side effects and tolerability.

The endorsement rate for participating in a therapeutic trial of ABMT + tDCS for the entire group was 75% and did not change between arms (ABMT + real tDCS: 78.6% ABMT + sham tDCS: 76.9%). This contrasts with what was reported in a study with people with BED (Flynn et al. 2024), in which real tDCS was associated with stronger endorsement than sham. It may reflect differences in the acceptability of the delivery method, that is, whereas Flynn et al.'s trial was fully remote (Flynn et al. 2024), ENTER, required multiple in‐person visits. However, despite high initial endorsement, no participant from the sham arm opted for open‐label real tDCS upon study completion due to time commitment, lack of perceived benefit, or finding the intervention tedious. Variations in treatment experiences and expectations about neuromodulation could also have increased participants' perceived burden, for example, the association of tDCS with more invasive procedures (electroconvulsive therapy and lobotomy) (Grycuk et al. 2021).

### 4.2 Clinical Outcomes Related to Eating Behaviours

Food craving traits were reduced in both groups, with small effect sizes favouring the ‘ABMT + sham tDCS’ group. However, relative to baseline, no significant changes in FCQ‐T scores were observed (T0), suggesting ABMT alone could have contributed to reductions in cravings, without benefit coming from tDCS. Some other studies have also not detected an effect of tDCS on craving, particularly in individuals in the normal weight range (Sedgmond et al. 2019; Beaumont et al. 2021) and with a low susceptibility to overconsumption and food cravings (Beaumont et al. 2021). In contrast, in individuals with BED, tDCS reduced food cravings (Burgess et al. 2016), with a meta‐analysis and systematic reviews indicating moderate effect sizes favouring active stimulation (Mostafavi et al. 2020; Dalton et al. 2017).

TDCS effects on reducing food craving may depend on sex (favouring females), food category preferences, and cognitive traits (Goldman et al. 2011; Kekic et al. 2014; Ray et al. 2017). In this respect, the FCQ‐T may lack the sensitivity of a behavioural task such as the Food Photo Craving Test, for detecting nuanced changes in craving (Kekic et al. 2016; Burgess et al. 2016; Ray et al. 2017). The FCQ‐T provides a global measure for food cravings without differentiating food categories, whereas, the Food Photo Craving Test can reveal changes in cravings for specific food categories (e.g., reduction in cravings for sweet but not savoury foods), (Kekic et al. 2016; Burgess et al. 2016; Sedgmond et al. 2019; Ray et al. 2017). Placebo or expectancy effects could also have contributed to the small reductions in both arms. For example, a study with 74 adults who were overweight or with obesity, found that expectation about treatment allocation, namely the participant's belief about their group assignment, was able to reduce food craving and eating, without an additional effect of tDCS alone (Ray et al. 2017). Finally, ABMT itself has been reported to reduce food cravings (Brockmeyer et al. 2015b; Schmitz and Svaldi 2017). Future trials should include behavioural measures, such as the Bogus Food Taste Test (as originally included in the ENTER protocol), to improve food craving assessment and elucidate underlying mechanisms.

Change scores with medium effect sizes favoured a reduction in binge eating episodes in the ‘ABMT + real tDCS’ arm, with participants showing a significant decrease in average binge episodes from baseline. This is consistent with research combining a somewhat different form of cognitive training to the one used here and tDCS in individuals with BED (Flynn et al. 2024; Giel et al. 2017), which demonstrated a progressive and delayed effect, observed at follow‐ups of 6 weeks and 3 months, respectively. In contrast, a pilot RCT of real versus sham ABMT in individuals with bulimia nervosa or BED found no significant benefits of real over sham ABMT, with both groups showing significant reductions in binge eating over time (Brockmeyer et al. 2019). Given the potential role of time as a delayed effect or as a confounding variable, trials should consider an extended follow‐up period and include a waiting list control arm. In addition, binge eating episodes were self‐reported via the EDE‐Q, which is susceptible to subjectivity. However, it is of note that 43% of participants in the ‘ABMT + tDCS’ group and 28.6% of participants in the ‘ABMT + sham tDCS’ arm reported more than 10 episodes of binge eating over the last 28 days, highlighting the need to screen participants for BED and to consider BED diagnosis as a stratification criterion in participants with SMI.

At baseline, participants in the ‘ABMT + real tDCS’ arm showed an average approach bias to food and those in the ‘ABMT + sham tDCS’ arm avoidance bias towards food when assessed using the SRCT. However, F‐AAT results indicate an initial approach bias towards food in both arms. Following the intervention, a small effect size favoured the ‘ABMT + real tDCS’ arm in reducing these scores, with moderate within‐groups effect size. Reductions in approach, avoidance, and approach‐avoidance biases measured by the SRCT favoured the ‘ABMT + real tDCS’ group, with small‐to‐medium effect sizes. Within‐groups analyses showed participants receiving active stimulation had a significant reduction in SRCT‐assessed approach‐avoidance bias and increase in SRCT‐avoidance bias, with medium‐to‐large effect sizes. One possible explanation for the inconsistency of the results of SRCT and F‐AAT at both baseline and post‐treatment, could be the task order. While performing F‐AAT first could have primed participants for SRCT, it could also have contributed to fatigue or reduced engagement during the SRCT task.

### 4.3 Secondary Clinical Outcomes

No effects of tDCS on schizophrenia‐related symptoms were observed, consistent with Pondé et al. (2017). Real tDCS in the present study did not promote positive changes in mood. In fact, reductions in change scores for depression, stress and anxiety favoured the ‘ABMT + sham tDCS’ arm. Per‐protocol analyses further showed that a significant and large effect size favoured the ‘ABMT + sham tDCS’ arm in reduction in PANSS‐6 change score. Within‐groups analyses revealed significant cognitive improvements in both arms, while mood, particularly DASS total and anxiety scores, was significantly reduced only in the ‘ABMT + sham tDCS’ arm. Rather than demonstrating a potential clinical efficacy of the ‘ABMT + sham tDCS’ intervention in improving psychological symptoms, these findings may indicate a selection effect, in which, participants who completed the study protocol were likely those with lower schizophrenia symptoms, and better cognition and mood. In addition, the results may be influenced by other factors, such as placebo effects arising from participant expectations and the consistency of the researcher‐participant relationship (Cao et al. 2021; Bernstein and Brown 2017). Future studies should explore whether longer brain stimulation protocols or alternative delivery formats (e.g., remote or inpatient) can better isolate and elucidate the therapeutic efficacy.

### 4.4 Limitations and Future Directions

The study has some limitations. However, to our knowledge, this is the first RCT exploring the combination of ABMT and tDCS to address eating behaviours related to AIWG. The significant reductions in binge eating support the feasibility and safety of this approach. The findings provide a foundation for future research, with effect‐size estimates informing sample sizes for future trials. They also suggest that cognitive training when combined with brain stimulation may have a potential synergistic effect in improving eating behaviours in individuals with SMI. Future studies should seek to optimise brain stimulation protocols and to assess their longer‐term effects in larger, more diverse populations to confirm clinical utility, as well as the optimal timing for intervention once antipsychotic treatment begins.

This feasibility study was not powered to detect statistical group differences. While use of sham tDCS as a control allowed for meaningful comparisons, absence of a placebo control for the ABMT component, limits conclusions about its independent effects. Future studies should use a four‐arm design, including a sham ABMT condition and waiting‐list group. Self‐reported measures for height and eating behaviours, while standard in the field, introduce potential bias. This was mitigated by use of validated instruments, but future studies should incorporate objective measures, for example, digital tracking or biomarkers. The study had a relatively small sample size, with missing data handled through pairwise deletion. This minimised data loss. Future studies should employ more robust methods, for example, multiple imputation. The number of intervention sessions was based on previous research, that is, balancing feasibility with evidence. However, more sessions may have led to greater effects.

Although inclusion criteria did not specify illness stage, the recruited sample had a long mean illness duration of 12.9 years (SD = 8.8) and an average BMI of 32.5 kg/m^2^ (SD = 6.4), suggesting that AIWG was already well‐established among participants. Targeting individuals at the start of their treatment will be imperative to assess the effectiveness of the combined approach in preventing or minimising AIWG. Future studies would benefit from follow‐up periods of 3–6 months to assess longer‐term outcomes. Recruitment challenges during the COVID‐19 pandemic and limitations associated with the logistics of attending a hospital in central London on seven occasions may have affected the generalisability of the findings. Expanding recruitment across a wider range of services and regions and offering travel compensation would facilitate the participation of people showing a variety of sociodemographic and symptoms profiles. It is of note that the study achieved 93% of its target sample size, demonstrating feasibility despite these challenges. Strengthening the relationship between clinicians and potential participants may enhance trust and improve enrolment. This could be achieved, for example, by facilitating introductions between the research team and patients and being open to answer questions about the intervention. Recruitment in familiar environments, such as service sites or waiting rooms, could foster a more supportive atmosphere, to help address concerns participants might have. Additionally, conducting the study in such familiar locations, with a known commute and staff, could improve participant retention and recruitment.

## 5 Conclusion

The initiation of antipsychotic therapy is a critical clinical window for early intervention to mitigate long‐term metabolic and psychiatric consequences. ABMT combined with tDCS is acceptable and may help reduce binge eating in patients on antipsychotic medication. A reduction in approach bias to food and an increase in avoidance bias to food, followed by fewer binge eatings in the real tDCS group, suggest a synergistic effect between cognitive training and brain stimulation. A larger randomised controlled trial is recommended and required to validate these findings and confirm their consistency in between‐group comparisons.

## Funding

This work is supported by funding from the National Institute for Health Research (NIHR) Biomedical Research Centre (BRC) at South London and Maudsley NHS Foundation Trust (SLaM) and King's College London. Mariana Lopes, Fiona Gaughran, Iain Campbell and Ulrike Schmidt receive salary support from the BRC. FG is, in part, funded by the NIHR Applied Research Collaboration South London (NIHR ARC South London) at King's College Hospital NHS Foundation Trust, by the Medical Research Council [MR/Z503563/1] as part of Hub for Metabolic Psychiatry, one of the 6 Hubs comprising the UKRI Mental Health Platform [MR/Z000548/] and by the Maudsley Charity. Gemma Gordon was funded by a BRC PhD studentship. The funder was not involved in the study design and writing of this trial protocol paper and was not involved in the collection and analysis of data, nor the writing of the study report. The views expressed are those of the author(s) and not necessarily those of the NHS, the NIHR or the Department of Health.

## Ethics Statement

The protocol version 1 dated 04/04/2019 was approved by the Oxford B Research Ethics Committee (REC, reference number: 19/SW/0095) and is a registered clinical trial under the International Standard Randomised Controlled Trial Number (ISRCTN) registry (registration number: ISRCTN13280178). The written informed consent of all participants was collected by the investigators before performing any study‐specific procedure.

## Conflicts of Interest

The authors declare no conflicts of interest.

## Supporting information


**Supporting Information: S1** Feasibility outcomes of a randomised controlled trial (RCT) of approach bias modification training (ABMT) combined with real versus sham (placebo) transcranial direct current stimulation (tDCS) in people taking antipsychotics.
**Supporting Information: S2** Reported side effects in randomised controlled trial (RCT) of approach bias modification training (ABMT) combined with real versus sham (placebo) transcranial direct current stimulation (tDCS) in people taking antipsychotics.
**Supporting Information: S3** Per‐protocol analysis showing mean change scores for clinical outcome measures from post‐treatment (T1)—baseline (T0) and from follow‐up (T2)—baseline (T0), including within‐groups and between‐group effect sizes (Cohen's *d* with 95% confidence intervals).

## Data Availability

The data that support the findings of this study are available from the corresponding author upon reasonable request.
