# Supplementary material for: Combining Cognitive Training and Brain Stimulation: A Novel Approach to Address Disordered Eating and Antipsychotic‐Induced Weight Gain in People With Severe Mental Illness
Source: Early Interv Psychiatry. 2026 Oct 26;20(11):e70275. doi: 10.1111/eip.70275 (PMC13697232; doi:10.1111/eip.70275)
Supplement: Supplementary file 1 [file EIP-20-0-s001.docx]

**Supplementary material 1**. Feasibility outcomes of a randomised controlled trial (RCT) of approach bias modification training (ABM) combined with real versus sham (placebo) transcranial direct current stimulation (tDCS) in people taking antipsychotics.

**Supplementary material 2**. Reported side effects in randomised controlled trial (RCT) of approach bias modification training (ABM) combined with real versus sham (placebo) transcranial direct current stimulation (tDCS) in people taking antipsychotics.

**Supplementary material 3.** Per-protocol analysis showing mean change scores for clinical outcome measures from post- treatment (T1) - baseline (T0) and from follow-up (T2) - baseline (T0), including within-groups and between-group effect sizes (Cohen’s d with 95% confidence intervals).

*Note:* Post-assessment/ follow-up scores minus baseline scores. *Significant difference at the p < 0.05 level for CIs and in paired t-tests. Abbreviations (in alphabetical order): ABMT, attention bias modification training; BMI, body mass index; DASS-21, Depression, Anxiety and Stress Scale – 21 items; BIS-11, Barratt Impulsiveness Scale; EDE-Q, Eating Disorders Examination Questionnaire; F-AAT, Food Approach/Avoidance Task; FCQ-Tr, Food Craving Questionnaire-Trait reduced version; MoCA, Montreal Cognitive Assessment; n, number of observations, PANSS-6, Positive And Negative Syndrome Scale; SD, standard deviation; SRCT, Stimulus-Response Compatibility Task; tDCS, transcranial direct current stimulation.
